# Comparison of Differentiation of Induced Pluripotent Stem Cells and Bone-Marrow Mesenchymal Stem Cells to Osteoblast: Osteogenesis versus Pluripotency

**Published:** 2016-05-01

**Authors:** T. Foroutan

**Affiliations:** Department of Animal Biology, Faculty of Biological Sciences, Kharazmi University, Tehran, Iran

**Keywords:** Induced pluripotent stem cells, Mesenchymal stromal cells, Gene expression profiling, Osteogenesis, Xite transcript, mouse [Supplementary Concept], Genes, myc, sox2 protein, xenopus [Supplementary Concept]

## Abstract

**Background:**

Derivation of induced pluripotent stem cells (iPSCs) from various adult somatic cells through over-expression of pluripotent genes could allow for the unlimited autologous supply in regenerative medicine. On the other hand the generation of various progenitors from bone-marrow mesenchymal stem cells (MSCs) is justly well established.

**Objective:**

In this study we compared the expression level of pluripotent genes *oct4, c-myc, sox-2, nanog, klf4* and *lin28* in iPSCs and MSCs derived from bone marrow. Also the potential of osteogenesis of iPSCs and bone-marrow MSCs were compared.

**Methods:**

We analyzed the expression level of *oct4, sox-2, c-myc, klf4, nanog* and *lin28* genes in human MSCs derived from iPSCs and MSCs by cell culture and real-time PCR. Also the expression level of *osteocalcin* and *osteopontin* in both groups were evaluated.

**Results:**

We found that the expression of osteogenic markers in differentiated iPSCs to osteoblast were higher than bone-marrow MSCs. While the levels of pluripotency genes *oct4, c-myc* and *klf4* in iPSCs were significantly (p<0.05) higher than bone-marrow MSCs, MSCs showed higher expression of *sox-2, nanog* and *lin28* compared with iPSCs (p=NS).

**Conclusion:**

It seems that the higher expression of *osteopontin* and *osteocalcin* in MSCs compared to iPSCs may be due to other factors (besides pluripotency) required for differentiation of stem cells to osteoblast.

## INTRODUCTION

Two types of stem cells are currently recognized: adult stem cells and embryonic stem cells (ESCs). Adult stem cells are harvested from different tissue sources and variously called multipotent mesenchymal stromal cells or mesenchymal stem cells [1-5]. Mesenchymal stem cells (MSCs) could differentiate into osteoblast, chondroblast, cardiomyocyte, or even cells of non-mesodermal derivation including hepatocytes and neurons [6]. Although bone-marrow MSCs are originally isolated from bone marrow, similar populations have been reported in other tissues such as adipose and umbilical cord blood tissue. Adult stem cells have limitations in their application because they cannot be propagated indefinitely in culture; number of these cells also decreases with aging. There is evidence that these cells may exhibit reduced proliferation and differentiation with aging [13-17]. ESCs are considered to be pluripotent stem cells and are derived from the inner cells mass. These cells are capable of differentiation into any cell types. In contrast to adult stem cells, ESCs can be cultured indefinitely while maintaining their pluripotency [18-20]. Because of ethical concerns association with the application of ESCs in regenerative medicine, there is paucity of information regarding their potential applications for tissue regeneration. On the other hand, Yamanaka and Takahashi managed to reprogram the somatic cells to pluripotent ESC-like cells by over-expression of transcription factors *oct4, sox-2, klf-4, c-myc, lin28* and *nanog* [21]. Stem cells obtained from this method is named “iPSCs.” They closely resemble ESCs because they restore a genome associated with a pluripotent marker. There are reports of attempts to generate osteoblast and chondroblast progenitors from ESCs and iPSCs [22].

In this study, we compared differentiation of iPSCs and bone-marrow MSCs into osteoblast using a monolayer approach. Osteoblast markers found in our *in vitro* samples were carefully analyzed. We also attempted to correlate expression of pluripotency markers *oct4, c-myc, sox-2, nanog, klf4* and *lin28* in iPSCs and MSCs before differentiation into osteoblast. 

## MATERIALS AND METHODS

Cell Culture

*Mesenchymal Stem Cells from Bone Marrow*

Human MSCs from the bone marrow aspirates were obtained from the iliac crest of healthy donors aged 25–35 years; the cells were collected in a syringe containing 10,000 IU heparin to prevent coagulation. The mononuclear cell fraction was isolated by Ficoll density gradient centrifugation (d 5 1.077 g/cm^3^; Biochrom, Berlin, Germany). In brief, mononuclear cells were plated in expansion medium at a density of 10^5^ cells/cm^2^ in tissue culture flasks (Nunc, Wiesbaden, Germany) coated with 10 ng/mL fibronectin (Sigma, Deisenhofen, Germany). The expansion medium consists of 58% Dulbecco’s Modified Eagle’s MediumdLow Glucose (DMEM-LG, Cambrex, Apen, Germany) and 40% MCDB201(Sigma), 2% fetal calf serum (FCS; StemCell Technologies, Vancouver, BC, Canada), supplemented with 2 mM L-glutamine, 100 U/mL Pen/Strep (Gibco, Eggenstein, Germany), 1% insulin transferrin selenium, 1% linoleic acid bovine serum albumin, 10 nM dexamethasone, 0.1 mM L-ascorbic acid-2-phosphate (all from Sigma), platelet-derived growth factor, and epidermal growth factor (10 ng/mL) (R&D Systems, Wiesbaden, Germany). On reaching 80% confluency, cells were trypsinized with 0.25% trypsin/1 mM EDTA (Invitrogen, Karlsruhe, Germany) and replated at about 9000 cells/cm^2^. Cells were expanded for 2–6 passages. The plastic adherent cell fraction was reseeded at a density of about 9000 cells/cm^2^. Cells were expanded for 2–6 passages. iPSCs were purchased from Royan Institute Quantitative real-time PCR: RNA of treated and non-treated MSCs, and iPSCs stem cells were extracted using Trizol reagent (Invitrogen) according to the manufacturer’s protocol. RNA was analyzed with quantitative real-time PCR (qPCR).

Melting curve analyses and PCR product sequencing were performed to verify primer specificities. RT-PCR was repeated at least three times using the following conditions. Each of the reaction mixtures contained 10 µL of SYBR Green master mix (Applied Biosystems), 5 pM each of forward and reverse primers and 5 µL of 100 times diluted cDNA. To synthesize cDNA, 1 µg of total RNA was used. The relative expression levels of each gene was determined with the 2−ΔΔCt method. The primer sequences used for qPCR are mentioned in Table 1.

**Table 1:** The sequence of primers

Gene	Forward	Reverse
*oct4*	5′-GAAACCCACACTGCAGATCA-3′	5′-GGTTACAGAACCACACTCG-3′
*sox-2*	5′-TGCTGCCTCTTTAAGACTAGGAC-3′	5′-CCTGGGGCTCAAACTTCTCT-3′
*nang*	5′-AGATGCCTCACACGGAGACT-3’	5′-TTTGCGACACTCTTCTCTGC-3′
*c-myc*	5′-CACCAGCAGCGACTCTGA-3′	5′-GATCCAGACTCTGACCTTTTGC-3′
*lin28*	5’-GGCAGTGGAGTTCACCTTTAAGA-3’	5′-AGCTTGCATTCCTTGGCATGATGA-3′
*GAPDH*	5′- ATGGGGAAGGTGAAGGTCG-3′	5′- GGGGTCATTGATGGCAACAATA-3′
*klf4*	5′-GGGAGAAGACACTGCGTCA-3′	3′-GGAAGCACTGGGGGAAGT-5′
*Osteocacin*	5′-TACAGACGAGGACATCAC-3′	5′-TCTACAACCAGCATATCTTC-3′
*Osteopontin*	5′-ATGAGAGCCCTCACACTCCTC-3′	5′-CCCAGCCATTGATACAGG-3′

In Vitro Osteogenic Assay

We seeded and expanded 5–10×10^4^ iPSCs and also MSCs per 6-well plates until nearly confluent. BMSC medium was then supplemented with dexamethasone + Ascorbic acid + β-glycerophosphate (mineralization medium) that was changed two or three times per week for 4–6 weeks, when signs of mineralization were visible under bright-field microscopy. Wells were fixed with fresh 4% formaldehyde for 1 hour, rinsed in double-distilled H_2_O (ddH_2_O), then incubated with 1% alizarin red S (weight per volume, with 97% ddH_2_O and 2% ethanol [volume per volume]) for 5 minutes. Excess stain was rinsed away with five changes in ddH_2_O. Each line but one was analyzed in triplicate [21]. Each test was repeated three times.

## RESULTS

**Figure 1:**
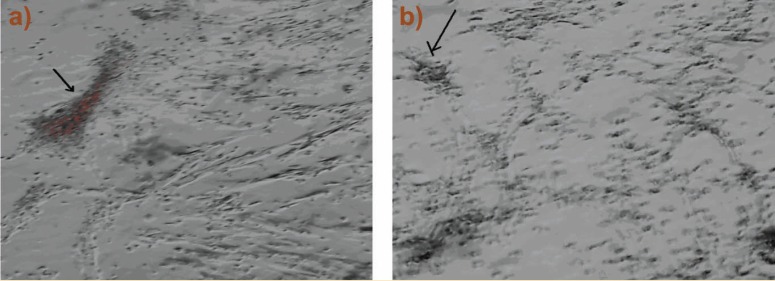
Phase contrast microscopy image of MSCs (a) and iPSCs (b) differentiated into osteoblast

**Figure 2:**
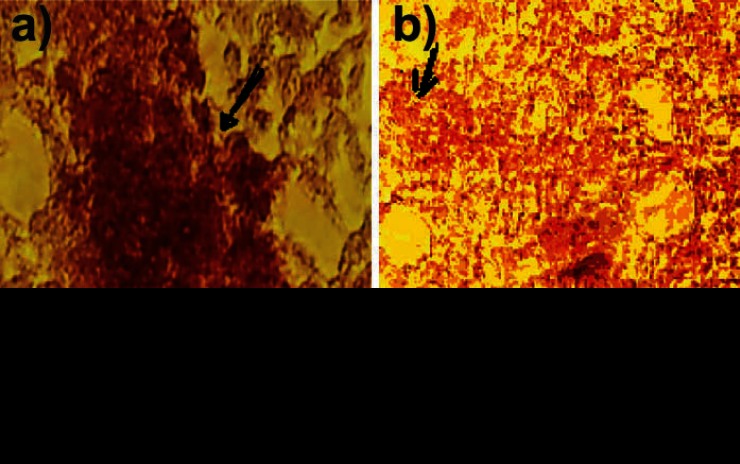
Light microscopy image of alizarin red stained MSCs and iPSCs (b) differentiated into osteoblast

**Figure 3:**
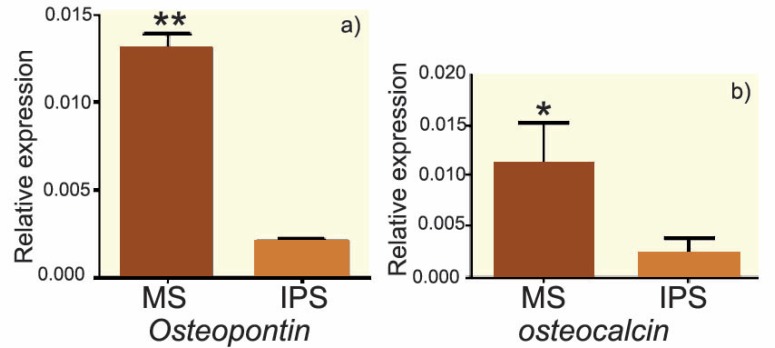
Real-time PCR results of *osteocalcin* (a) and *osteopontin* (b) genes in iPSCs and MSCs differentiated into osteoblast. *p<0.05

**Figure 4:**
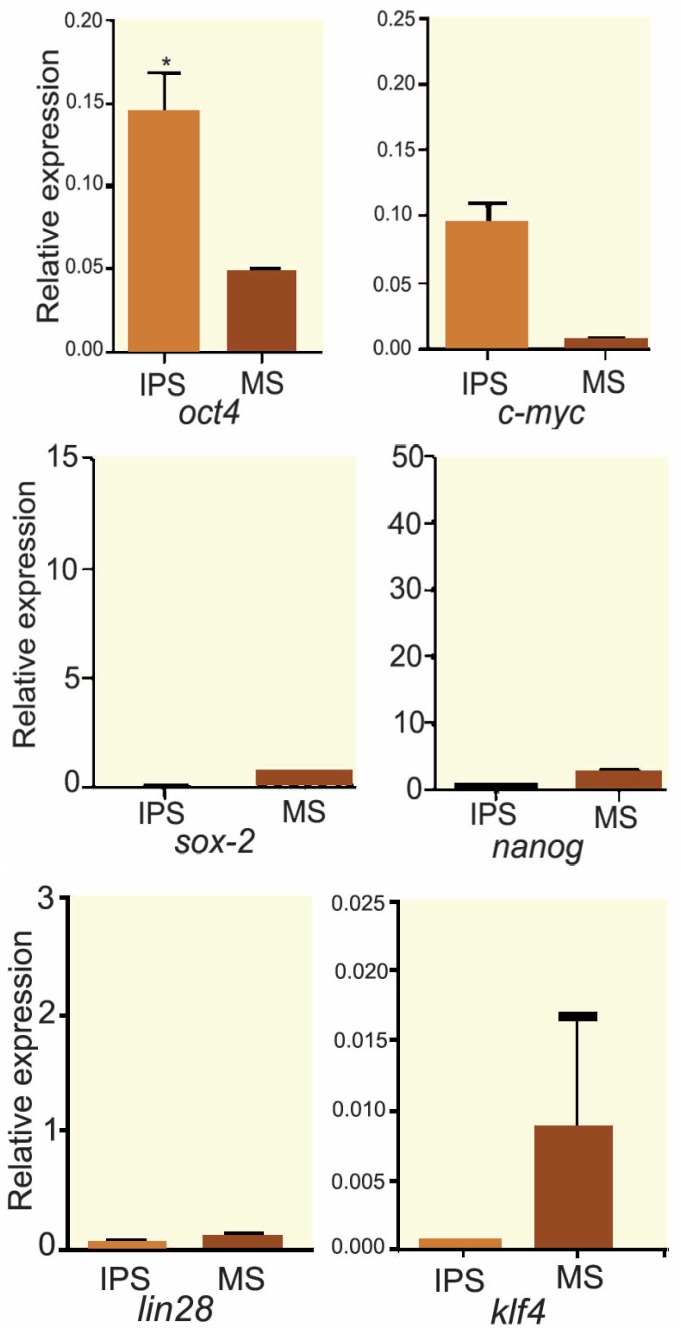
Comparative real-time PCR analysis of *oct4, sox-2, c-myc, nanog, klf4 *and *lin28* genes expression in MSCs and iPSCs. Expression of *oct4 *and* c-myc *and* klf4 *genes in iPSCs is significantly higher than that in MSCs. A significantly (p<0.05) lower level of *sox-2, nanog, *and* lin28* genes expression was detected in iPSCs compared with MSCs significantly

The results of alizarin red staining showed that the mineralization process, where a reddish purple mass was observed in some areas of culture, indicated a positive trend of osteogenesis in human bone-marrow MSCs. The mass was observed in both groups. Figures 1 and 2 show that the rate of osteogenesis in MSCs group increased significantly (p<0.05) compared with another group. Figure 3 indicates the expression of *osteocalcin* and *osteopontin* in both groups. Expression of *osteocalcin* and *osteopontin* genes in MSCs group was significantly (p<0.05) higher than that in iPSCs group. In the present study, we compared the expression of six genes in human MSCs and iPSCs. Our data showed a significantly (p<0.05) higher expression of *oct4, c-myc* and *klf4* in iPSCs compared with that in another group (Fig 4). MSCs expressed significantly lower level of *oct4* and *c-myc* pluripotent markers than that in iPSCs group. In contrast, expression of *sox2, nanog,* and* lin28* was higher in MSCs compare with iPSCs group (p=NS) (Fig 4).

## DISCUSSION

In this study the osteogenesis potential of bone-marrow MSCs and iPSCs reprogrammed from skin fibroblast were compared. We evaluated the expression of osteogenic markers, oseopontin and *osteocalcin*, and showed that the expression of osteoblast markers in MSCs was higher than that in iPSCs. 

Our results showed that the expression of some pluripotent markers such *oct4* and *c-myc* in iPSCs was significantly (p<0.05) higher than bone-marrow MSCs. On the other hand, our results showed the expression of some pluripotent markers such as *sox-2, nanog* and *lin28* in bone-marrow MSCs were more than that in iPSCs (p=NS).

*Oct4* and *c-myc* are widely accepted as markers for pluripotent stem cells such as ESCs and iPSCs [22]. The expression of *oct4 *has already been reported in several adult somatic cells [23]. *Oct4* expression in differentiated cells challenges its role as a pure stem cell marker [24]. Tai and colleagues reported that *oct4* expression in somatic cells is restricted to small populations of multipotent cells with high self-renewal capacity, namely the adult stem cells [23]. Recently, researchers succeeded to induce pluripotent stem cells from primary human fibroblasts by only *oct4* and *sox-2* reprogram factors [25]. In the present research, *oct4*, as the most important pluripotent factor, expressed in both MSCs and iPSCs. It seems that a higher expression of *osteopontin* and *osteocalcin* in MSCs compared with iPSCs may be attributed to other factors (besides pluripotency) required for differentiation of stem cells to osteoblast.

Ratajczak and colleagues suggested* oct4* is an embryonic transcription factor that occurs at low concentrations in somatic cells [26]. Tsai and colleagues reported that over-expression of only *oct4* and *klf4* genes is sufficient to induce reprogramming without exogenous or endogenous *c-myc* [27]. We found that both cells studied expressed *oct4* gene and that the expression of *oct4* transcriptional factor was significantly higher in iPSCs than bone-marrow MSCs. Izadpanah, *et al*, concluded that *oct4* is not specific to pluripotent stem cells [28]. In keeping with their findings, our results also showed that *oct4* was not specific to pluripotent stem cells. One possible explanation could be that MSCs have some properties of pluripotent stem cells while they are being considered adult stem cells. We previously showed that MSCs derived from adipocyte tissue endogenously express high levels of *c-myc* [29]. Therefore, these cells can be reprogrammed into iPSCs merely by *oct4* expression. Our data showed that iPSCs expressed the main pluripotent stem cells markers, *oct4* and *c-myc* more than MSCs. The other possible explanation could be based on Bhartia hypothesis who asserts that the true stem cells in adult body tissues are the very small embryonic-like stem cells (VSELs), whereas MSCs are actually progenitor stem cells that arise by asymmetric cell division of VSELs [30]. The higher expression of osteogenesis markers in MSCs differentiated to osteoblast in comparison with iPSCs can indicate that in addition to pluripotent genes, other factors might also play a role in the osteogenesis differentiation.
